# Predicting stone free rate after retrograde intrarenal surgery using RIRS scoring system versus Resorlu Unsal stone score (RUSS)

**DOI:** 10.1080/20905998.2023.2252227

**Published:** 2023-10-16

**Authors:** Basheer N. Elmohamady, Mahmoud M. Farag, Hamouda W. Sherif, Ahmed EL Ghobashy, Mohamed A. AL Hefnawy

**Affiliations:** a Urology Department, Benha Faculty of Medicine, Benha University, Benha, Egypt; b professor of urology, Benha faculty of medicine, Benha university, Benha, Egypt

**Keywords:** RIRS, RUSS, SFR, scoring system, predictive value

## Abstract

**Background:**

To evaluate the predictive ability of the RIRS scoring system and the RUSS in predicting stone-free rate (SFR) after retrograde intrarenal surgery (RIRS).

**Methods:**

This prospective study was conducted on patients who underwent RIRS for kidney stones. Two scoring systems were used to determine the degree of procedure difficulty: the RIRS scoring system and the RUSS. We assessed the predictive ability of the two scoring systems utilizing receiver operating characteristic (ROC) analysis and calculated the sensitivity and specificity of each system. Additionally, we analyzed the association between the scoring systems and the stone-free outcome using a multivariate logistic regression model.

**Results:**

One hundred seventy-one patients were incorporated into this study with a mean age of 43 years, and 65.5% were male. The results showed a significant AUC of 0.868 for the RIRS score (*P* < 0.001, 95% CI = 0.813–0.924). The sensitivity and specificity were 72% and 93.7%, respectively. In contrast, the RUSS score revealed a non-significant unsatisfactory AUC of 0.480 (*P* = 0.660), with a 95% confidence interval ranging from 0.384–0.576.

**Conclusion:**

The RIRS scoring system showed a better predictive ability for SFR after RIRS than the RUSS. Additionally, RIRS was a significant predictor of SFR, controlling for age, gender, body mass index, and previous renal surgery.

## Background

Kidney stones are a widespread health condition, with a prevalence of 12% in men and 6% in women. It is estimated that kidney stones are more frequently occurring, and the disease is becoming a global burden on healthcare systems [1]. Therefore, accurate diagnosis, proper management, and follow-up are crucial for preventing complications and achieving a favorable outcome [2].

Recent advancements in endourological techniques and technologies, including flexible ureteroscopy (FURS), have revolutionized the handling of kidney stones [3–5]. The procedure is highly effective, safe, and has a lower complication rate than traditional surgical methods [6].

Despite the advances in the endourological management of kidney stones, there are still some challenges in foreseeing a stone-free outcome after the procedure. Various factors, comprising stone size, density, location, and patient-related factors, such as age, gender, and comorbidities, have been reported to affect stone-free outcomes. Therefore, a trustworthy scoring system to foresee the outcome after FURS is highly needed [7].

Several scoring systems have been made to anticipate stone-free outcomes after Percutaneous nephrolithotomy, comprising the RENAL score, S.T.O.N.E. score, and Guy’s stone score. These scoring methods have been proven reliable in different studies and have exhibited promising results in foreseeing a stone-free outcome [8,9].

Also many parameters and systems were used to decide the outcome of the stone free rate (SFR) after retrograde intrarenal surgeries (RIRS) as stone site, burden, hydronephrosis, but, when these parameters are used separately, they are not reproducible and do not give accurate idea about the outcome of SFR [9,10].

So scoring systems were developed by using different parameters.These Scoring systems which could predict the SFR and guide management decisions and to give concrete data for preoperative consultancy to patients and to determine the most suitable operation type [8–10].

However, the success of RIRS depends on various elements, including stone size and location, and patient-related factors. Therefore, it is definitive to have a reliable scoring system to predict SFR after RIRS [8].

However, the success of RIRS depends on diverse elements, comprising stone size and location, in addition to patient-related factors. Therefore, it is crucial to have a reliable scoring system to predict SFR after RIRS [11].

Resorlu Unsal Stone score (RUSS) is a commonly used scoring system that predicts the SFR after ureteroscopic lithotripsy [12]. However, its predicting ability remains unclear [12].

A new scoring system, called the RIRS scoring system, has been developed recently. It comprises stone density, renal infundibulopelvic length, renal infundibulopelvic angle, and stone burden. RIRS scoring system has demonstrated promising results in foreseeing SFR after RIRS [13].

This study aimed to compare the predictive ability of the RIRS scoring system and RUSS in predicting SFR after RIRS.

**RIRS Scoring System**

**Table:** 

	Points
Stone diameter ≤10 mm	1
RIL ≤25 mm	1
Stone density ≤ 1000 HU	1
Calculus outside lower pole	1
Stone diameter > 10 - ≤ 20 mm	2
RIL >25 mm	2
Stone density > 1000 HU	2
Calculus in lower pole	2
RIPA >300	2
Stone diameter >20 mm	3
Calculus in lower pole	3
RIPA ≤300	3
Total Score Range	4–10

## Methods

One hundred seventy-one patients who visited the Urology Department of Benha University Hospital and underwent RIRS for kidney stones were prospectively examined. FURS managed all patients. Preoperative CT scans and postoperative imaging were performed on all patients for comparison. Information about patients’ characteristics (demographics, previous ipsilateral urinary tract surgery, and preoperative placement of ureteral stents), the stone factors (total stone burden, stone number, and stone density), and the renal factors (anatomical abnormalities, stone location in a lower pole, number of calyceal involvement) were collected. The stone-free outcome was defined as a residual fragment ≤4 mm. NCCT was revised preoperatively and classified each case using RUSS, considering four factors (1 point for each): stone size >20 mm; lower pole position with infundibulopelvic angle (IPA) < 45°; stone number in various calyces > 1; aberrant renal anatomy. The total score ranges from 0 to 4.

The RIRS Scoring system categorizes patients based on several factors, including stone density (HU), renal infundibulopelvic length (RIL), renal infundibulopelvic angle (RIPA), and stone burden (mm). The scoring system assigns points based on specific criteria. For stone diameter ≤10 mm, RIL ≤25 mm, stone density ≤ 1000 HU, and the location of the calculus outside the lower pole, one point is assigned. For stone diameter between > 10 - ≤ 20 mm, RIL >25 mm, stone density > 1000 HU, the existence of a calculus in the lower pole, and RIPA > 300, two points are assigned. Three points are given for stone diameter >20 mm, the existence of a calculus in the lower pole, and RIPA ≤ 300. The total score ranges from 4 to 10.

RIL was measured by calculating the distance from the stone furthest point to the renal pelvis midpoint. RIPA is the term used to describe the inferior angle resulting from the interaction of the lower calyx axis with the ureteropelvic axis.

### Registration and ethical considerations

The current research followed the declaration of Helsinki ‎for studies involving humans, and the Benha ‎University ethics committee approved it (Ms; 12.3.2022). All patients gave written consent before participation.

### Operative technique

All RIRS procedures were performed under general anesthesia and in a lithotomy position. Before the procedure, ureteral dilatation was performed by ureteral dilators up to 12–14 Fr. A 9.5/11.5 Fr ureteral access sheath (Cook Medical Bloomington, IN, USA) was inserted over the guidewire under fluoroscopy. For all cases, two guidewires were used, but only a safety wire was left outside the access sheath. Flexible URS was conducted using a 9.5 Fr (The LithoVue™ System-Boston Scientific). Stones were managed with a holmium: YAG laser. After the procedure, a JJ stent was inserted.

### Sample size calculation

The sample size was calculated using the MedCalc software version 18.2.1 based on a previous estimate of the AUC of the RIRS score of 0.817. The minimum sample size calculated was 140 patients (93 stone-free and 47 with residual). Alpha and power were adjusted at 0.05 and 0.8, respectively.

### Statistical analysis

Data management and statistical analysis were performed using version 28 of SPSS (IBM, Armonk, New York, United States). Quantitative data were checked for normality using the Kolmogorov-Smirnov test and direct data visualization methods. Means and standard deviations or medians and ranges were employed to summarize quantitative data following normality. Numbers and percentages were employed to summarize a categorical set of data. With respect to normally and non-normally distributed numerical variables, the independent t-test or Mann-Whitney U test was employed to compare quantitative data between patients with and without residual stones. We compared categorical data using the Chi-square test. Linear by linear association was assessed using Cochrane Armitage test for trend. The performance of the RUSS and RIRS scores in foreseeing the stone-free outcome was evaluated using ROC analysis. Diagnostic indices and areas under the curve (AUC) with 95% confidence intervals were computed. Multivariate logistic regression analysis was employed to predict the stone-free outcome. Odds ratios with 95% confidence intervals were estimated. There were two sides to every single statistical test. Significant *P* values were defined as those less than 0.05.

## Results

### General and clinical characteristics

The mean age of the studied patients was 43 ± 15. Two-thirds (65.5%) were males. The mean BMI was 27.8 ± 4.2. One-third (35.7%) had associated co-morbidities, while 33.7% had previous renal surgeries. Two thirds of the patients (62.6%) were stone-free. Patients demonstrated significantly lower age (40 ± 15 vs. 48 ± 13, *P* < 0.001), BMI (27.1 ± 4.2 vs. 28.8 ± 3.8, *P* = 0.009), and comorbidities (29.9% vs. 45.3%, *P* = 0.042) compared to those with residual stones. No significant differences were reported regarding sex (*P* = 0.332) and previous renal surgery (*P* = 0.286) (Table 1).

**Table 1.** General and clinical characteristics of the studied patients according to stone-free status.

		Stone free		
	Total	Yes (*n* = 107)	No (*n* = 64)	P-value
**Age (years)**	43 ± 15	40 ± 15	48 ± 13	**<0.001**
**Sex**				
Males	112 (65.5)	73 (68.2)	39 (60.9)	0.332
Females	59 (34.5)	34 (31.8)	25 (39.1)	
**Body mass index**	27.8 ± 4.2	27.1 ± 4.2	28.8 ± 3.8	**0.009**
**Associated comorbidity**	61 (35.7)	32 (29.9)	29 (45.3)	**0.042**
**Previous renal surgery**	55 (33.7)	32 (30.8)	23 (39.0)	0.286
**Loin pain duration (months)**	1 (0.25–8)	1 (0.25–7)	1 (0.25–8)	0.673
**Stones size (mm)**	14 ± 4	13 ± 3	17 ± 4	**<0.001**
**Stone density (HU)**	1028 ± 329	817 ± 153	1380 ± 226	**<0.001**
**Multiple stones**	40 (23.4)	5 (4.7)	41 (54.7)	**<0.001**
**Lower pole stone**	50 (29.2)	17 (5.9)	33 (51.6)	**<0.001**
**RIPA (°)**	80 (20–160)	120 (44–160)	60 (20–150)	**<0.001**
**RIL (mm)**	16 (2–38)	14 (10–30)	20 (2–38)	0.650
**Hydronephrosis**	58 (33.9)	35 (32.7)	23 (35.9)	0.666
**Abnormal renal Anatomy**	8 (4.7)	4 (3.7)	4 (6.3)	0.452
**RUSS**	1 (0–3)	1 (0–2)	1 (0–3)	0.600
**RIRS**	5 (2–10)	4 (2–7)	6 (4–10)	**<0.001**
**Complications**	49 (28.7)	31 (29.0)	18 (28.1)	0.906

Data are presented as mean ±SD, median (min-max), or number (percentage); RIPA: renal infundibulopelvic angle; RIL: renal infundibular length; RUSS: Resorlu Unsal stone score; RIRS: retrograde intrarenal surgery score. Significant P-values are marked in bold.

The median loin pain duration was 1 month, ranging from 1 week to 8 months. The mean stone size was 14 ± 4. The mean stone density was 1028 ± 329 HU. One-quarter (23.4%) had multiple stones. One-third had lower pole stones (29.2%). The median RIPA was 80°, ranging from 20–160, while the median RIL was 16 mm, ranging from 2–38 mm. One-third (33.9%) had hydronephrosis. Only 4.7% had abnormal renal anatomy. Median RUSS was 1 (0–3), while the median RIRS 5 (2–10). Complications were reported in 28.7% (Table 1).

Stone-free patients had significantly lower stone size (13 ± 3 vs. 17 ± 4 mm, *P* < 0.001), stone density (817 ± 153 vs. 1380 ± 226 HU, *P* < 0.001), multiple stones (4.7% vs. 54.7%, *P* < 0.001), lower pole stones (5.9% vs. 51.6%, *P* < 0.001), and RIRS (median = 4 vs. 6, *P* < 0.001) as opposed to those with residual stones. In contrast, RIPA was significantly greater in stone-free patients (median = 120 vs. 60, *P* < 0.001) (Table 1).

Loin pain duration, hydronephrosis, abnormal renal anatomy, RUSS, and complications did not significantly differ (*P* = 0.673, 0.666, 0.452, 0.6, and 0.906, respectively) (Table 1).

### Stone free rate according to RUSS and RIRS scores

A significant linear by-linear association was reported between RIRS and SFR, with the SFR decreasing with the higher scores (*P* < 0.001). The RUSS score revealed a non-significant association (*P* = 0.725) (Table 2).

**Table 2.** Stone free rate according to the RUSS and RIRS score.

	Stone free n (%)	P-value
**RUSS**		
0 (*n* = 48)	24 (50)	.725
1 (*n* = 111)	81 (73)	
2 (*n* = 9)	2 (22.2)	
3 (*n* = 3)	0 (0)	
**RIRS**		
2 (*n* = 1)	1 (100)	<.001
4 (*n* = 80	76 (95)	
5 (*n* = 47)	21 (44.7)	
6 (*n* = 32)	8 (25)	
7 (*n* = 9)	1 (11.1)	
9 (*n* = 1)	0 (0)	
10 (*n* = 1)	0 (0)	

RUSS: Resorlu Unsal stone score; RIRS: retrograde intrarenal surgery score; Significant P-values are marked in bold.

### ROC analysis of RUSS and RISS scores to predict the stone-free outcome

**Figure 1.**
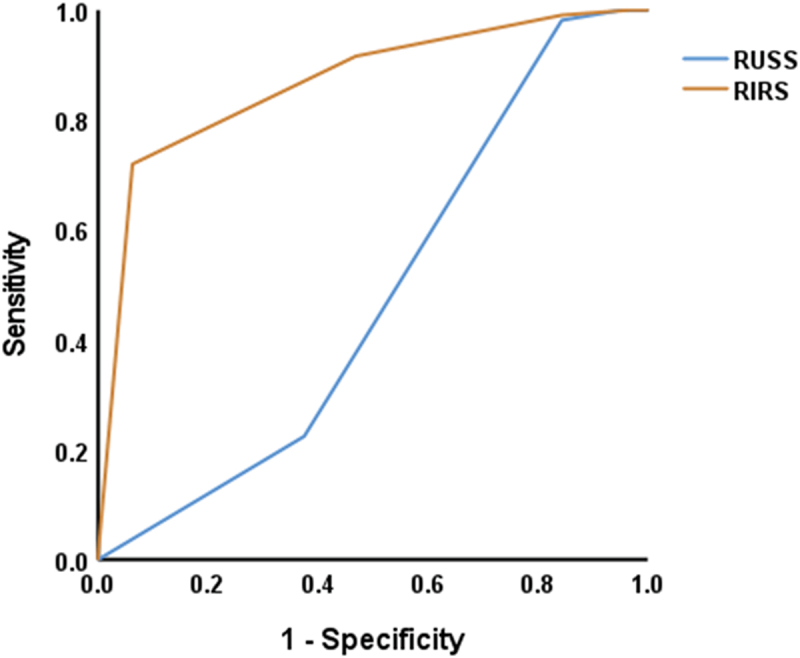
ROC analysis of RUSS and RISS score to predict the stone-free outcome.

To determine the accuracy of RUSS and RIRS scores in foreseeing a stone-free outcome, a ROC analysis was carried out. The results showed a significant AUC of 0.868 for the RIRS score (*P* < 0.00, 95% CI = 0.813–0.924). Sensitivity and specificity were 72% and 93.8%, respectively. The positive and negative predictive values were 95.1% and 66.7%, respectively. The overall accuracy was 80.1%. In contrast, the RUSS score revealed a non-significant unsatisfactory AUC of 0.480 (*P* = 0.660, 95% CI = 0.384–0.576). The sensitivity and specificity were 22.4% and 62.5%, while the PPV and NPV were 50% and 32.5%, respectively. The overall accuracy was 37.4% (Figure 1). Additionally, poor agreement was observed between RUSS and RIRS score as measured by Kappa statistic (Kappa = −0.089).

### Prediction of stone-free outcome using RUSS and RIRS

A multivariate logistic regression analysis was done to predict the stone-free outcome. Predictors were selected clinically. To avoid potential multicollinearity that will result from adding all the selected predictors together, as some variables were correlated, each predictor was evaluated separately, controlling for potential confounders, including age, sex, BMI, co-morbidities, and surgical history. Additionally, RIPA was measured only for those with lower pole stones, and if added to other predictors will result in elimination of all cases without RIPA values from the model, leading to a great loss of information.

The results revealed that controlling for age, gender, BMI, comorbidities, and previous renal surgery, one millimetre increase in the stone size was associated with 29.8% reduction of the stone-free outcome (OR: 0.702, 95% CI: 0.616–0.801, *p* < .001). One HU increase in the stone density was associated with 3% reduction of the stone-free outcome (OR: 0.966, 95% CI: 0.948–0.984, *p* < .001). Presence of multiple stones was associated with 94.4% reduction of the stone-free outcome (OR: 0.056, 95% CI: 0.019–0.160, *p* < .001). Presence of lower pole stones was associated with 86.8% reduction of the stone-free outcome (OR: 0.132, 95% CI: 0.058–0.302, *p* < .001). High RIRS score > 4 was associated with 98.1% reduction of the stone-free outcome (OR: 0.019, 95% CI: 0.005–0.069, *p* < 0.001). RIPA > 90 was associated with 20 times increase of the stone-free outcome (OR: 20.792, 95% CI: 3.071–288.982, *p* = 0.003) (Table 3).

**Table 3.** Multivariate logistic regression analysis to predict the stone-free outcome.

	OR (95% CI) *	P-value
**Stone size (mm)**	0.702 (0.616–0.801)	<.001
**Stone density (HU)**	0.966 (0.948–0.984)	<.001
**Stone number (ref: single)**		
Multiple stones	0.056 (0.019–0.160)	<.001
**Stone site (ref: other sites)**		
Lower pole	0.132 (0.058–0.302)	<.001
**RIPA (ref: ≤ 90)**	1.07 (1.021–1.120)	.004
RIPS >90	29.792 (3.071–288.982)	.003
**RIRS score (ref: ≤ 4)**		
RIRS >4	0.019 (0.005–0.069)	<.001

*Adjusted for age, gender, BMI, comorbidities, and previous renal surgery; OR: Odds ratio; 95% CI: 95% confidence interval; RIPA: renal infundibulopelvic angle; RIRS: retrograde intrarenal surgery score; Significant P-values are marked in bold.

## Discussion

Our study aimed to evaluate the predictive ability of the RIRS scoring system and the RUSS in predicting stone-free rate (SFR) after retrograde intrarenal surgery (RIRS). In this prospective study on patients undergoing RIRS for kidney stones, two scoring systems (RIRS and RUSS) were employed to assess procedure difficulty, and the relationship between the scoring systems and stone-free outcome was examined among 171 participants, with a mean age of 43 years and 65.5% being male.

Regarding the clinical characteristics, the current research reported that the stone-free patients had significantly lower stone size, stone density, multiple stones, lower pole stones, and RIRS than those with residual stones. Stone size and location are known to be significant predictors of SFR after FURS [14]. In line with these findings, Kim et al. retrospectively reviewed the records of 237 patients who underwent URSL for ureteral stones and declared that stone diameter, length, and density significantly affected the stone-free rate [15]. Other studies highlighted that stone-free patients had smaller stone sizes, lower stone density, and fewer multiple and lower pole stones than those with residual stones, buy residual stones can be easily treated with repeat URS [16]. However, Perlmutter et al. evaluated the impact of stone location on success rates of flexible URS. A total of 86 renal stones were treated, and the stone-free rates for upper-, middle-, and lower-caliceal stones were 100%, 95.8%, and 90.9%, respectively. They concluded that stone location does not significantly affect stone clearance rates [17].

Interestingly, our study found no significant association between hydronephrosis, abnormal renal anatomy, RUSS score, and complications with the stone-free outcome. Confirming our study, a recent study by Napitupulu et al. found that there was no statistically significant relationship between hydronephrosis and stone clearance (P-value = 0.310) [18]. Additionally, Ergani et al. in their study to examine whether the presence of hydronephrosis in the patient has an effect on the stone-free rates in FURS applications determined that age, gender, side, number, size and the Hounsfield Unit of the stone, and the presence of hydronephrosis and its degree did not affect the stone-free rate [19]. Also, Satyanarayan et al. found that different age groups, gender, BMI, urine culture, hydronephrosis were correlated with the post-operative stone clearance and observed that stone clearance was not significantly associated with them [20].

The present study findings revealed that lower pole and multiple stones were significant predictors of the stone-free outcome, consistent with other studies. A systematic review and meta-analysis by Kallidonis et al. showed that the management of lower pole stones by percutaneous nephrolithotripsy or retrograde intrarenal surgery achieved stone-free status over a short period and minimal number of sessions and for stones smaller than 10 mm, retrograde intrarenal surgery is more efficient in comparison to shock wave lithotripsy [21].

The current study concluded that the RIRS score significantly predicted stone-free outcomes. SFR decreased with higher RIRS scores, which is compatible with previous studies [11,22] showing that the RIRS score significantly predicts the stone-free outcome after FURS.

The current study reported that the RUSS score did not predict the stone-free outcome. Contrasting our findings, Tufano et al. found that RUSS was identified as the only predictive score for SFR (OR: 0.32; *p* = 0.002). Selmi et al. in a pooled comparison of different nephrolithometric scores showed that RUSS was the best predictor of SFR (OR: 0.45) [23]. Additionally, Erbin et al. assessed and compared the applicability of the Resorlu-Unsal Stone Score (RUSS) and the Modified Seoul National University Renal Stone Complexity (S-ReSC) score for flexible ureterorenoscopy (f-URS). They retrospectively analyzed the hospital files of 339 patients (168 men and 171 women) out of 719 patients who had been treated with f-URS for kidney stone. In the logistic regression analysis, musculoskeletal deformity, stone size, and the RUSS were identified as independent predictive factors affecting stone-free status (*p* < 0.001, 0.014, and 0.048, respectively), and among these parameters, the RUSS had the highest predictive capability (AUC = 0.65, 95% CI = 589, 721) [24]. The variation in results between our study and these studies may be attributed to some differences, as each study have included patients with different demographic profiles, stone compositions, sample sizes and anatomical variations, that could affect the predictive capability of the RUSS score [24].

The present study assessed the accuracy of the RIRS and RUSS scores in foreseeing the stone-free outcome using ROC analysis. The results revealed that the RIRS score had a significant AUC of 0.868, with a sensitivity of 72% and specificity of 93.7%. In contrast, the RUSS score had an unsatisfactory AUC of 0.480. These findings are consistent with Sanguedolce et al. and De et al. showing that the RIRS score is a better predictor of the stone-free outcome after FURS than the RUSS score [25,26]. However, Tufano et al. reported that RUSS is a simple scoring system with high efficacy and accuracy in foreseeing postoperative SFR following RIRS as RUSS registered an AUC of 0.76 [27]. Similarly, Sfoungaristos et al. externally validated RUSS estimating an AUC of 0.70 [12].

Interestingly, the results from a meta-analysis comparing the predictive ability of the most used scoring systems for SFR has not revealed any superiority of one scoring tool over another [22]. However, the high heterogeneity between studies and variables between the scoring systems make difficult to statistically generalize these findings.

Finally, this study had some limitations: Firstly, we included a relatively small sample size of 171 patients. Secondly, the study did not evaluate the effects of different surgical techniques on the stone-free rate, which could have impacted the outcomes. Thirdly, the long-term outcomes of patients were not evaluated. Lastly, the RIRS procedure heavily relies on the operator’s expertise and has a potential for bias. Therefore, future prospective multi-center studies should be conducted over a large sample size to investigate the durability of the stone-free state over time.

## Conclusions

The SFR decreased with the higher RIRS scores, while the RUSS score showed a non-significant association with the stone-free outcome. Furthermore, ROC analysis demonstrated that the RIRS score had higher accuracy in foreseeing stone-free outcomes than the RUSS score. These results support the usage of the RIRS score as a valuable tool in foreseeing the success of treatment and can help make more informed decisions regarding the management of renal stones. However, additional research is essential to verify these findings.

## List of abbreviations

**Table:** 

95% CI	95% confidence interval
AUC	area under the curve
BMI	body mass index
fURS	flexible ureteroscopy
RUSS	Resorlu Unsal stone score
IPA	infundibulopelvic angle
OR	Odds ratio
RIL	renal infundibulopelvic length
RIPA	renal infundibulopelvic angle
RIRS	retrograde intrarenal surgery
ROC	receiver operating characteristic
RUSS	Resorlu Unsal stone score
SFR	Stone-free rate

## Data Availability

The datasets used and analyzed for the current work are available upon reasonable request from the corresponding author.
